# Class I/Class II HLA Evolutionary Divergence Ratio Is an Independent Marker Associated With Disease-Free and Overall Survival After Allogeneic Hematopoietic Stem Cell Transplantation for Acute Myeloid Leukemia

**DOI:** 10.3389/fimmu.2022.841470

**Published:** 2022-03-04

**Authors:** Anne-Marie Daull, Valérie Dubois, Hélène Labussière-Wallet, Fabienne Venet, Fiorenza Barraco, Sophie Ducastelle-Lepretre, Marie-Virginie Larcher, Marie Balsat, Lila Gilis, Gaëlle Fossard, Hervé Ghesquières, Maël Heiblig, Florence Ader, Vincent Alcazer

**Affiliations:** ^1^ Hospices Civils de Lyon, Department of clinical Hematology, Lyon Sud hospital, Pierre-Bénite, France; ^2^ Laboratory of histocompatibility, Etablissement Français du Sang, Lyon, France; ^3^ Hospices Civils de Lyon, Immunology laboratory, Edouard Herriot Hospital, Lyon, France; ^4^ Centre International de Recherche en Infectiologie (CIRI), Inserm U1111, CNRS, UMR5308, Ecole Normale Supérieure de Lyon, Université Claude Bernard-Lyon 1, Lyon, France; ^5^ UR LIB “Lymphoma Immuno-Biology”, Université Claude Bernard Lyon I, Lyon, France; ^6^ Hospices Civils de Lyon, Department of infectious diseases, Croix-Rousse hospital, Lyon, France; ^7^ LegioPath team, CIRI INSERM U1111 CNRS UMR 5308, Lyon, France

**Keywords:** acute myeloid leukemia, hematopoietic (stem) cell transplantation (HCT), HLA Evolutionary divergence, graft-versus-host disease (GVHD), graft-versus-leukemia (GVL), immune reconstitution

## Abstract

Class I Human Leukocyte Antigen (HLA) evolutionary divergence (HED) is a metric which reflects immunopeptidome diversity and has been associated with immune checkpoint inhibitor responses in solid tumors. Its impact and interest in allogeneic hematopoietic stem cell transplantation (HCT) have not yet been thoroughly studied. This study analyzed the clinical and immune impact of class I and II HED in 492 acute myeloid leukemia (AML) recipients undergoing HCT. The overall cohort was divided into a training (n=338) and a testing (n=132) set. Univariate cox screening found a positive impact of a high class I HED and a negative impact of a high class II HED on both disease-free (DFS) and overall survival (OS). These results were combined in a unique marker, class I/class II HED ratio, and assessed in the testing cohort. The final multivariate cox model confirmed the positive impact of a high *versus* low class I/class II HED ratio on both DFS (Hazard Ratio (HR) 0.41 [95% CI 0.2-0.83]; p=0.01) and OS (HR 0.34 [0.19-0.59]; p<0.001), independently of HLA matching and other HCT parameters. No significant association was found between the ratio and graft-versus-host disease (GvHD) nor with neutrophil and platelet recovery. A high class I HED was associated with a tendency for an increase in NK, CD8 T-cell, and B cell recovery at 12 months. These results introduce HED as an original and independent prognosis marker reflecting immunopeptidome diversity and alloreactivity after HCT.

## Background

Allogeneic hematopoietic stem cell transplantation (HCT) is the only curative therapy for several malignant hematological diseases and acute myeloid leukemia (AML). The success of this therapy partly relies on the recognition of cancer cell antigens by alloreactive T cells, leading to the so-called graft-versus-leukemia (GVL) effect (1). When recognized antigens are present on normal tissue, this effect is counterbalanced by graft-versus-host disease (GVHD), one of the main sources of morbidity and death after HCT (2). Central to immune balance, Human Leukocyte Antigens (HLA) are key molecules responsible for antigen presentation to T cells. The importance of HLA matching is currently well established, resulting in HLA matched sibling donors being the primary choice of stem cell source to reduce the risk of GVHD through allo-recognition of foreign HLA molecules (3). In addition to HLA main alleles, recognition of minor-histocompatibility antigens which represent non-HLA self-peptides that can be recognized by allogeneic T-cells, is an additional source of alloreactivity (4, 5). However, the appreciation of genetic diversity through minor-histocompatibility antigen mismatching requires a costly additional genome-wide sequencing. To date, no other markers of T-cells alloreactivity exist to complement HLA matching.

HLA molecules present a high degree of polymorphism, the diversity of which can be evaluated by the HLA evolutionary divergence (HED) between the peptide-binding domains of HLA alleles. Polymorphism in HLA class I molecules is indeed a major determinant of the immune response mediated by CD8^+^ T-cells, as a more diverse repertoire allows the presentation of a more diverse set of antigens (6, 7). Accordingly, an increased polymorphism in HLA class I, as assessed by HLA heterozygosity, has been associated with an enhanced response to immune checkpoint inhibitors (ICI) in advanced cancer patients (8). More recently, Chowell et al. demonstrated that a high HED in class I alleles is strongly associated with ICI response due to a broader immunopeptidome diversity (9). HED impact has also been studied in the context of liver graft selection, and a high HLA class I diversity of the donor was found to be associated with a worse outcome, helping in graft selection (10). To date, the impact of class I and II HED has never been evaluated in HCT recipients from related and unrelated donors.

In this study, the clinical and immune impact of class I and II HED in recipients of allogeneic HCT for the treatment of AML was assessed. First, the impact of individual HED scores on disease free (DFS) and overall survival (OS) after HCT were investigated. Then, using stratified random sampling, different scores were compared on both a training and a testing set of 338 and 132 AML patients, respectively. Class I/class II HED ratio was found to be strongly predictive of both DFS and OS, independently of disease status, conditioning, graft source, and HLA matching. Finally, the impact of class I HED on immune reconstitution at 6 and 12 months after HCT was evaluated.

## Methods

### Patients and Study Design

This retrospective, single center, observational study was conducted among adult (≥18 years) AML patients who underwent allogeneic HCT at the hematology department of a French tertiary-care university hospital (*Hôpital Lyon Sud, Hospices Civils de Lyon*, Lyon, France) between January 2006 and December 2019. AML patients were treated according to the French standard-of-care based on the ELN 2010 recommendations updated in 2017 (11). Patients who died before day 14 after HCT were excluded from the study. Clinical endpoints were extracted from a prospectively maintained database. Three main periods were defined to appropriately account for treatment evolutions: 2006-2010, 2010-2015, and 2015-2019. The study was performed according to the local ethics committee guidelines (ID: 21_5581).

### Cohort Split

The total cohort was split into a training set and a testing set using stratified random sampling based on disease status, HCT number, conditioning, HLA matching, and HCT date. The cohorts’ equilibrium was then assessed by checking the overall distribution of these variables together with additional variables such as age, graft source, acute and chronic GVHD incidence and grade. Statistical comparisons of baseline characteristics between the two cohorts were eventually performed using the Student’s t-test for continuous variables, and the Chi-squared test for categorical variables.

### HLA Evolutionary Divergence

HLA typing was performed by the national blood bank center (*Etablissement Français du Sang*, Lyon, France) using a polymerase-chain reaction sequence-specific primer with 2-field resolution (and 1-field resolution for familial and cord blood grafts performed before 2014). HED was calculated using the Grantham distance, a metric representing the qualitative divergences between two protein sequences by considering physicochemical properties of amino-acids (12). The distance is calculated using the original formula:


$$Grantham\;Distance={\sum \left[\alpha {\left(c_{i}-c_{j}\right)}^{2}+\beta {\left(p_{i}-p_{j}\right)}^{2}+\gamma {\left(v_{i}-v_{j}\right)}^{2}\right]}^{1/2}$$

where *i* and *j* are the homologous amino-acids at a given position in the alignment and *c*, *p* and *v* their respective composition, polarity and volumes. *α, β* and *γ* represent constants. All these values can be retrieved from the original study (12). The Grantham distance between HLA proteins was calculated as previously described (9, 13). Briefly, protein sequences corresponding to the peptide-binding domain (exons 2 and 3 for class I and exon 2 for class II) of each HLA molecule referenced in the IMGT HLA database (14) were extracted using Ensembl annotations (15). Divergences between each allele represented by the Grantham distance were calculated using a custom Perl script published by Dr. Tobias Lenz (https://sourceforge.net/projects/granthamdist/files/) (13). Mean HED scores were calculated in both donors and recipients as the mean divergence between the two copies of HLA-A, -B, and -C for class I, and HLA-DRB1, -DPB1, and -DQB1 for class II (**Supplementary Figure S1A**).

### Multivariate Cox Screening

The impact of HED on DFS and OS was evaluated for each allele in a multivariate cox screening. Classical variables impacting DFS and OS were first identified in univariate analysis. Variables with an impact on both DFS and OS (FDR-adjusted p-value < 0.10) were included as controlling factors in the multivariate cox screening. Disease status, HCT number, conditioning, use of total body irradiation, HLA matching (identical sibling, 10/10 matched unrelated, 9/10 mismatched unrelated and haplo-identical donors), and graft source were retained for the final multivariate cox screening in which each HED variable was independently tested, controlling for all the confounding factors.

### Establishment of Scores and Final Multivariate Models

Four different scores were established based on the ratio of HED numerical values obtained from recipients and based on the observed clinical impact of each HED: class I/class II (score 1), class I + DRB1/DQB1 (score 2), class I + DPB1/DQB1 (score 3) and class I + DPB1/DQB1 + DRB1 (score 4). Final multivariate models were established using the class I/class II HED ratio with all the controlling variables. Backward stepwise regression was performed to eliminate non-significant variables.

### Immune Reconstitution

Immune parameters were retrieved from a prospective cohort study evaluating detailed lymphocyte subsets by routine flow cytometry from peripheral blood samples collected at 6 and 12 months after HCT. Data were available for 96 patients from 2014 to 2019 (see **Supplementary Methods**). Multivariate logistic regression analysis was performed using the median class I HED to determine the impact of a high *versus* low class I HED on immune reconstitution.

### Endpoints

The primary endpoint of the study was to evaluate the impact of HED on DFS and OS after HCT. Secondary objectives were to assess the impact of HED on acute and chronic GVHD, and on hematologic and lymphocyte subsets’ recovery after HCT.

### Statistical Analysis

Continuous variables are described as medians (interquartile range [IQR]), and categorical variables as count (%). The descriptive table function from the StatAid R package was used for descriptive analysis (16). Univariate and multivariate Cox models were setup using the survival R package (17). Missing values were not imputed. All statistical analyses were performed using R statistical software v4.0.5 (18). P-values were adjusted using the Benjamini-Hochberg correction (False Discovery Rate) when multiple testing was performed in the screening phase. A non-adjusted p-value cutoff of 0.01 was considered for significancy in the final multivariate model.

## Results

### Baseline Characteristics of Patients and Descriptive Analysis

**Figure 1:**
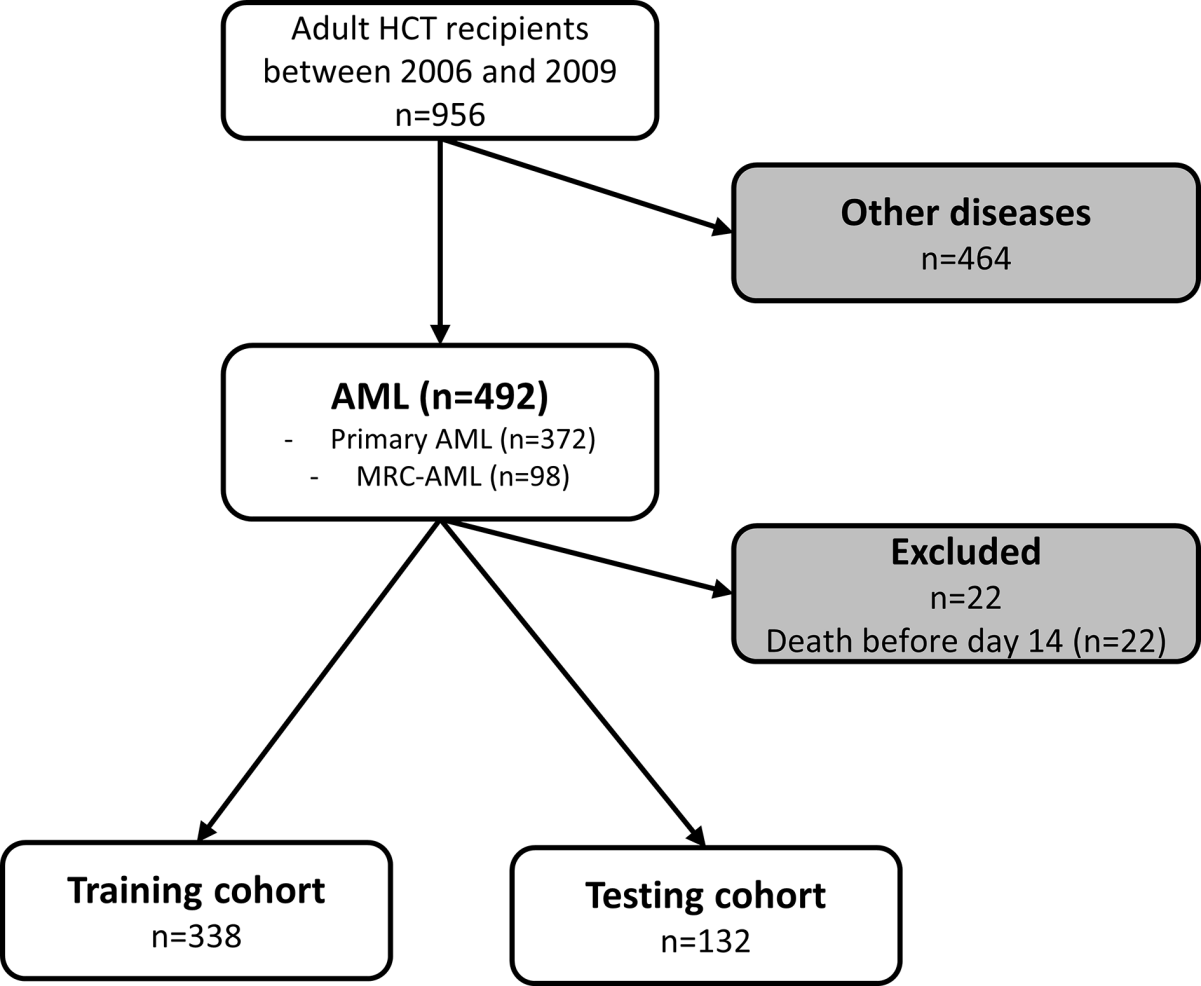
Flow chart of patients included in the study. AML, acute myeloid leukemia; HCT, hematopoietic cell transplantation; MDS, myelodysplastic syndrome; MRC, myelodysplasia related change.

Between 2006 and 2019, 492 AML patients underwent allogeneic HCT in our institution. Twenty-two patients were excluded for early death before day 14 (**Figure 1**). Among the 470 remaining patients, 372 (79.2%) presented a primary AML and 98 (20.9%) presented an AML with myelodysplasia-related changes (MRC-AML). The median age was 50.3 years [IQR 38.2-59.1], and there were 214 (45.5%) females. Most patients were in complete remission (n=350, 75%) and received a single HCT (n=429, 91.3%). Peripheral blood cells (PB) was the most used graft source (n=320, 68.1%). The median follow-up of censored patients was 23.1 months [IQR 9.7-86.1] (**Table 1**).

**Table 1:** AML patient’s baseline characteristics.

Variable		Whole cohort	Training set	Testing set	p-value
Age	years, [Median (IQR)]	50.34 [38.16-59.14]	49.84 [37.87-58.95]	52.12 [39.3-59.26]	0.33
Gender	Female [2]	214 (45.53)	155 (45.86)	59 (44.7)	0.90
	Male [1]	256 (54.47)	183 (54.14)	73 (55.3)	
Diagnosis	AML	372 (79.15)	271 (80.18)	101 (76.52)	
	MRC-AML	98 (20.85)	67 (19.82)	31 (23.48)	
Disease_status	Early	350 (74.95)	246 (73.43)	104 (78.79)	
	Intermediate	48 (10.28)	38 (11.34)	10 (7.58)	0.43
	Advanced	69 (14.78)	51 (15.22)	18 (13.64)	
HCT_number	First	429 (91.28)	309 (91.42)	120 (90.91)	1.00
	Second_or_higher	41 (8.72)	29 (8.58)	12 (9.09)	
HCT_date_cat	2006-2010	94 (20)	69 (20.41)	25 (18.94)	0.92
	2010-2015	190 (40.43)	135 (39.94)	55 (41.67)	
	2015-2019	186 (39.57)	134 (39.64)	52 (39.39)	
Graft_source	Bone Marrow	93 (19.79)	66 (19.53)	27 (20.45)	0.91
	Cord Blood (CB)	57 (12.13)	40 (11.83)	17 (12.88)	
	Peripheral Blood (PB)	320 (68.09)	232 (68.64)	88 (66.67)	
HLA_match	Identical sibling	176 (37.45)	126 (37.28)	50 (37.88)	0.96
	Matched Unrelated	145 (30.85)	104 (30.77)	41 (31.06)	
	Mismatched Unrelated	131 (27.87)	94 (27.81)	37 (28.03)	
	Haplo-identical	18 (3.83)	14 (4.14)	4 (3.03)	
Conditioning	MAC	188 (40)	134 (39.64)	54 (40.91)	0.88
	RIC	282 (60)	204 (60.36)	78 (59.09)	
TBI	No [1]	338 (71.91)	241 (71.3)	97 (73.48)	0.72
	Yes [2]	132 (28.09)	97 (28.7)	35 (26.52)	
aGVHD	No	200 (42.74)	145 (43.15)	55 (41.67)	0.13
	Grade I [1]	113 (24.15)	89 (26.49)	24 (18.18)	
	Grade II [2]	76 (16.24)	47 (13.99)	29 (21.97)	
	Grade III [3]	45 (9.62)	30 (8.93)	15 (11.36)	
	Grade IV [4]	34 (7.26)	25 (7.44)	9 (6.82)	
cGVHD	No	315 (67.02)	227 (67.16)	88 (66.67)	1.00
	Yes	155 (32.98)	111 (32.84)	44 (33.33)	
cGVHD_extent	Extensive [2]	59 (46.83)	41 (45.56)	18 (50)	0.80
	Limited [1]	67 (53.17)	49 (54.44)	18 (50)	
Follow-up	months, [Median (IQR)]	23.08 [9.67-86.13]	22.3 [9.39-92.52]	24.23 [13.13-71.72]	

Data are presented as n (%) for dichotomous variables or median (interquartile range [IQR]) for continuous variables. Statistical comparisons of baseline characteristics between the training and testing sets were performed using the Student’s t-test for continuous variables, and the Chi-squared test for categorical variables. There were no significant differences in baseline characteristics between the two cohorts (p-value range from 0.13 to 1.00).

aGVHD, acute graft-versus-host disease; cGVHD, chronic graft-versus-host disease; AML, acute myeloid leukemia; HCT, hematopoietic cell transplantation; HLA, Human leukocyte antigens; MAC, myeloablative conditioning; MDS, myelodysplastic syndrome; MRC, myelodysplasia-related change; TBI, total body irradiation.

Using the Grantham distance, the HED for both recipient and donor class I and II HLA was calculated (see methods). Mean HED scores followed a roughly similar distribution between recipients and donors, with a significantly lower mean HED for class I compared to class II alleles (mean (sd) of 6.97 (1.7) *versus* 9.57 (3.3), t-test p-value = 5.66x10^-46^ in recipients and mean (sd) of 6.99 (1.82) *versus* 9.66 (3.46), t-test p-value = 3.81x10^-44^ in donors; **Supplementary Figure S1B**). No significant correlations were found between the HED of the different alleles (**Supplementary Figure S1C**). As expected, HED scores where perfectly correlated between donors and recipients for identical sibling and 10/10 HLA matched donors, except for -DPB1 in this latter case (**Supplementary Figure S1D**). Regarding 9/10 HLA mismatched donors, HED scores showed inferior but still strong correlations between donors and recipients (Pearson’s R of 0.5, 0.6, 0.66, 0.83 and 0.8 for HED-A, -B, -C, -DQB1 and -DRB1 respectively, FDR adjusted p-values < 0.01, **Supplementary Figure S1D**).

### HED Impact on DFS and OS

**Figure 2:**
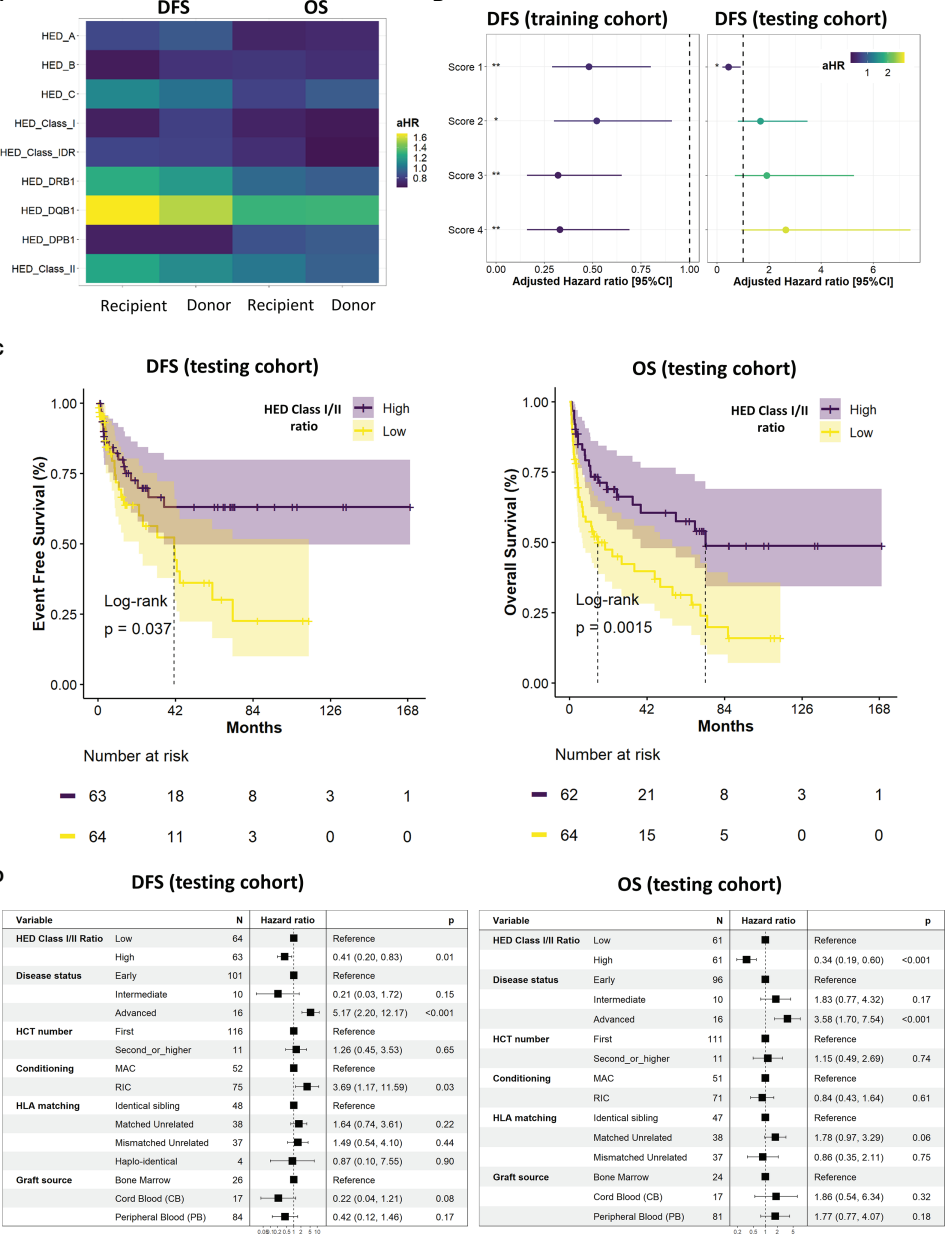
Survival analyses and ratio establishment. **(A)** Impact of individual allele HED on overall (OS) and disease-free (DFS) survival for both recipients and donors. Adjusted hazard ratio (HR) were calculated using a multivariate cox model controlling for disease status, HCT number, conditioning, use of total body irradiation, HLA matching, and graft source. **(B)** Evaluation of the impact on DFS of the four established scores in the training (left) and testing (right) cohorts. Adjusted HR were calculated using a multivariate cox model controlling for disease status, HCT number, conditioning, use of total body irradiation, HLA matching, and graft source. FDR-adjusted p-values significance is represented (*: < 0.05, **: < 0.01, ***: < 0.001). **(C)** Kaplan Meier curves of DFS (left) and OS (right) according to the median class I/class II HED ratio (score 1) in the testing cohort. **(D)** Final multivariate cox models of DFS (left) and OS (right) according to the median class I/class II HED ratio (score 1), disease status, HCT number, conditioning, HLA matching, and graft source in the testing cohort. A non-adjusted p-value cutoff of 0.01 was considered for significancy in the final multivariate model. DFS, disease free survival; HED, HLA evolutionary divergence; CI, confidence interval; HCT, hematopoietic cell transplantation; HLA, Human leukocyte antigens; HR, hazard ratio; OS, overall survival.

The impact of HED on DFS after allogeneic HCT was then evaluated. Regarding correlations between HED scores and the importance of recipient’s HLA molecules in antigen presentation, only recipient’s HED scores were kept for survival analyses. The cohort split based on stratified random sampling resulted in a training set composed of 338 patients and a testing set of 132 patients, with no significant differences in baseline characteristics found between the two groups (**Table 1**). Multivariate cox screening of each individual recipient HED scores together with previously identified controlling variables (disease status, HCT number, conditioning, use of total body irradiation, HLA matching, and graft source) found a global tendency for a better DFS with higher mean HLA class I and HLA-DPB1 HED, and a tendency for a worse DFS with higher mean HLA class II, and more specifically with HLA-DQB1 HED (**Figure 2A**). The positive impact of class I HED was also found on OS and was quite similar between donors and recipients, reflecting HLA matching.

To gain further insights regarding HED impact and to account for its different effects using a unique variable, four scores consisting in recipient ratios of HED were established: class I/class II (score 1), (class I + DRB1)/DQB1 (score 2), (class I + DPB1)/DQB1 (score 3), and (class I + DPB1)/(DQB1 + DRB1) (score 4). Whilst the four scores had a significant positive impact on DFS in the training cohort, this significant impact was only observed for score 1 in the testing cohort (**Figure 2B**). The final Kaplan-Meier curves (**Figure 2C**) and multivariate cox models (**Figure 2D**) confirmed the positive impact of a high *versus* low recipient class I/class II HED ratio (score 1) in the testing cohort, on both DFS (adjusted hazard Ratio (aHR) 0.41 [95%CI 0.2;0.83]; p=0.01) and OS (aHR 0.34 [95%CI 0.19;0.60; p<0.001). In the overall cohort, class I/class II HED ratio ranged from [0-2.3] (low tercile group) to [4.5-6.8] (high tercile group).

### Class I/Class II HED Ratio Association With GVHD and Neutrophil Recovery

**Figure 3:**
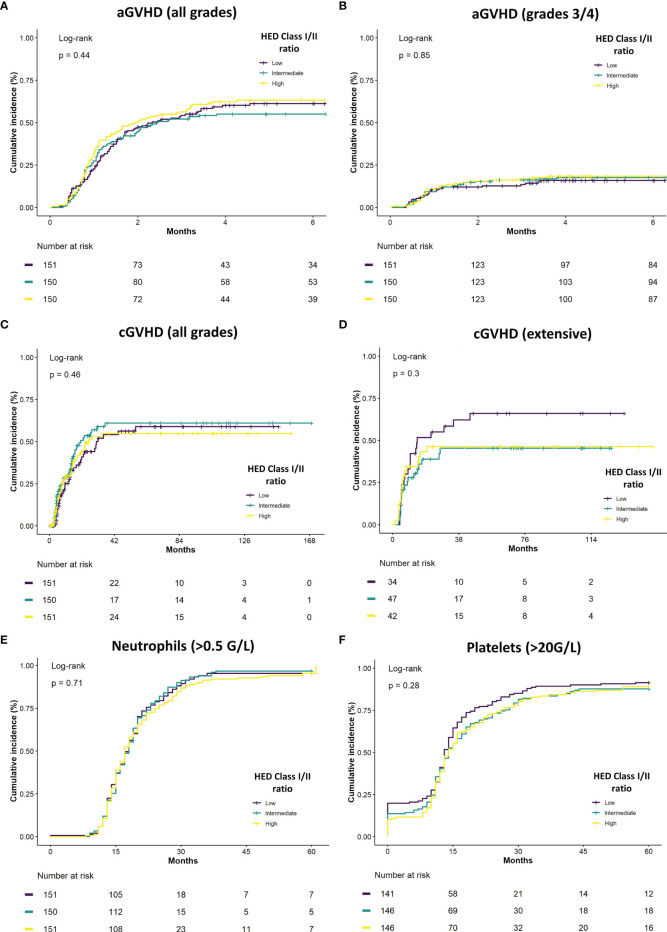
Class I/class II HED ratio association with GVHD and neutrophil recovery. **(A)** Cumulative incidence of overall acute GVHD (aGVHD) according to class I/class II HED ratio terciles in the overall cohort. **(B)** Cumulative incidence of severe grade 3/4 aGVHD according to class I/class II HED ratio terciles in the overall cohort. **(C)** Cumulative incidence of overall chronic GVHD (cGVHD) according to class I/class II HED ratio terciles in the overall cohort. **(D)** Cumulative incidence of severe grade 3/4 cGVHD according to class I/class II HED ratio terciles in the overall cohort. **(E)** Cumulative incidence of neutrophil recovery (> 0.5 G/L) according to class I/class II HED ratio terciles in the overall cohort. **(F)** Cumulative incidence of platelet recovery (> 20 G/L) according to class I/class II HED ratio terciles in the overall cohort. aGVHD, acute graft versus host disease; cGVHD, chronic graft versus host disease; HED, HLA evolutionary divergence.

The association between class I/class II HED ratio and different key clinical parameters was then explored. No significant association was found between HED and all grades and grades 3/4 acute GVHD (**Figures 3A, B**) nor with all grades and extensive chronic GVHD (**Figures 3C, D**). These results were confirmed in a multivariate model of individual class I and II HED scores and class I/class II HED ratio, controlling for disease status, HLA matching, conditioning, and graft source (**Supplementary Figure S2A, B**). Regarding graft function, neither neutrophil nor platelet recovery was found to be significantly associated with HED, in neither univariate (**Figures 3E, F**) nor multivariate analyses (**Supplementary Figure S2C**).

### Immune Reconstitution

**Figure 4:**
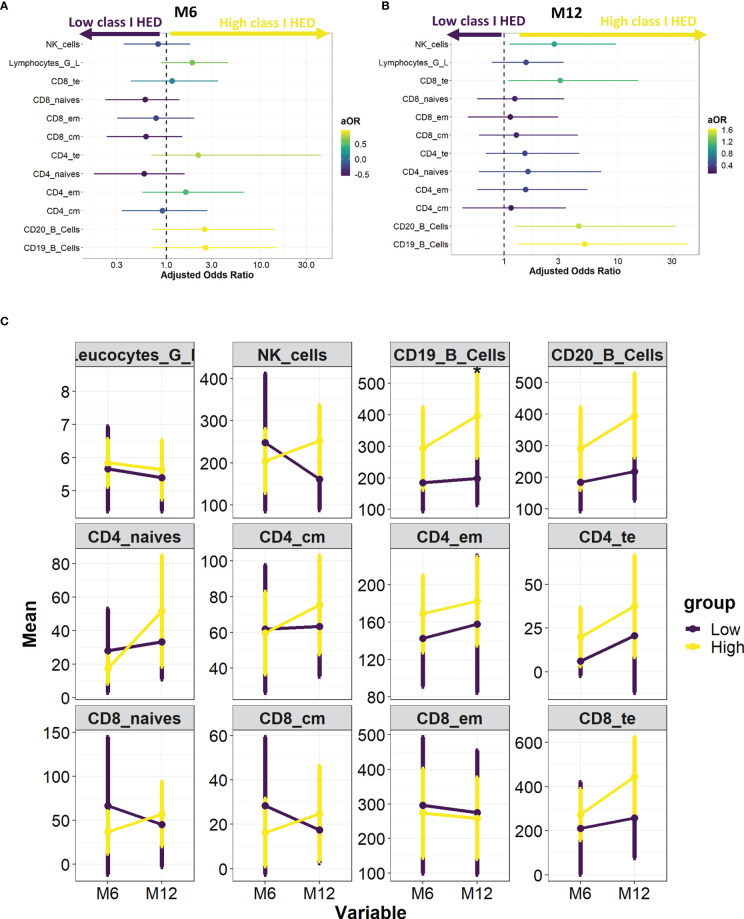
HED impact on immune reconstitution at 6 and 12 months post-HCT. **(A)** Association between high *versus* low median class I HED and lymphocyte subset recovery at 6 months. Odds Ratio were calculated using a multivariate logistic regression model controlling for HLA matching and conditioning. **(B)** Association between high *versus* low median class I HED and lymphocyte subset recovery at 12 months. Odds Ratio were calculated using a multivariate logistic regression model controlling for HLA matching and conditioning. **(C)** Evolution of total leucocyte and lymphocyte subset counts according to median class I HED at 6 and 12 months post-HCT. CM, central memory; EM, effector memory, HCT, hematopoietic cell transplantation; HED, HLA evolutionary divergence; NK, natural killer; TE, terminal effector.

As a higher class I HLA diversity is theoretically associated with a broader antigenic repertoire, we investigated whether the overall favorable prognosis associated with class I HED translated into differences in the immune reconstitution profile after HCT. Using prospectively collected flow-cytometry data from 96 AML patients at 6 and 12 months after HCT, the association between a high *versus* low class I HED and lymphocyte subset recovery was evaluated using a multivariate logistic regression model controlling for HLA matching and conditioning. Whilst no statistically significant associations were found at 6 and 12 months after HCT, a tendency for an increased recovery of Natural Killer (NK) cells, CD8+ T_TE_ cells, and CD19+/CD20+ B-cells at 12 months was observed in the high *versus* low class I HED group (**Figures 4A–C**).

## Discussion

While advances in HLA typing have improved HLA matching and enhanced graft attribution (19, 20), relapse after HCT remains frequent and is still subject to uncertain outcomes. The balance between GVL and GVHD is a key point governing HCT success. However, few parameters exist to appreciate alloreactivity, particularly in HLA-matched settings. In this study, we show that a high *versus* low class I/class II HED ratio is an independent factor associated with improved DFS and OS after HCT in AML patients.

The GVL effect is dependent on the presentation of leukemia-associated antigens to effector T-cells through HLA molecules (21–24). In line with the divergent allele advantage hypothesis (25, 26), Pierini & Lenz (13) showed that an enhanced diversity between HLA alleles increases the range of presented peptides and their subsequent recognition by effector T-cells. Thereafter, Chowell et al. (9) proposed to use HED, a quantification of physicochemical sequence divergences between HLA alleles calculated using the Grantham distance, to correctly appreciate such functional diversity. Patients with a high class I HED showed an improved OS after ICI therapy in melanoma and non-small cell lung cancer, a higher class I HED reflecting a broader immunopeptidome diversity (8, 9).

Using a large cohort of 492 AML patients, the impact of both recipient and donor class I and II HED on DFS and OS after HCT was explored here. Roerden et al. (27) have already reported a positive impact of a high HLA class I and DR HED on OS after HLA-matched HCT in AML patients. However, no validation cohort was used and some key confounding factors such as HCT number, graft source, and conditioning were not accounted for. Moreover, only HCT from sibling donors was studied and HED on other HLA class II alleles were not calculated. In the current study, a training and a testing set were used and no optimal cutoff selection was applied to avoid overfitting and enable the identification of stable predictive factors. Using this approach, a high class I HED and a low class II HED, as represented by the class I/II HED ratio, were found to be independent factors associated with improved DFS and OS after HCT in AML recipients. Importantly, this effect was independent of HLA matching and graft characteristics. These results suggest that HED could be an interesting metric even in a HLA mismatched transplant setting, providing an additive marker of GVL in addition to the main allogenic effect associated with the mismatched HLA.

As suggested by the univariate screening, the positive impact of class I HED appears to be mainly driven by HLA-A and -B diversity. Among HLA class I loci, HLA-B locus is the oldest and the most polymorphic. As reported by Hughes et al. (28) its diversity results particularly from interallelic recombination occurring in the peptide-binding region (exons 2-3). HLA-C appears to be less divergent than HLA-A and -B with a shorter distance in terms of both nucleotide differences (29) and Grantham distance (9, 27, 30). This is mainly explained by the more recent evolution of HLA-C, which has emerged from the duplication of the HLA-B gene (31). With a lower cell surface density, HLA-C molecules are in fact thought to have evolved to assume a role in NK cell regulation by providing an inhibitory signal recognized by killer immunoglobulin-like receptors (32–35).

The impact of class I HED on immune reconstitution after HCT had never been studied before. While no significant association was found between class I HED and hematological recovery and immune reconstitution at 6 and 12 months after HCT, a tendency for an improved recovery of NK cells, CD8+ T_TE_ cells, and CD19+/CD20+ B-cells at 12 months was observed. Whilst the improved T-cell recovery could easily be associated with a broader immunopeptidome diversity, the link with B and NK cells is more difficult to explain. One hypothesis would be that NK cells recovery could be partly driven by -C HED and a higher class I HED could favor T_FH_ development and B-cells recovery. The small number of patients used for the immune reconstitution analysis may have impacted the power of this secondary analysis, and further studies using data prospectively collected will be needed to highlight a possible link between higher class I HED and lymphocyte subset recovery.

Unexpectedly, this study found a negative impact of class II HED on DFS and OS. This negative impact appeared to be mainly driven by -DQB1 HED, while a tendency for a better outcome was observed with a higher -DPB1 HED. These different impacts led us to consider four different scores to better define a potentially clinically relevant ratio, but only the ratio using the overall class II HED was found to be consistent and significant in the testing cohort. Similarly to what is known on HLA class I mismatch, the negative impact of HLA-DR disparity is well established (20, 36). Although HLA-DQ mismatch is linked through strong linkage disequilibrium with HLA-DR, its tolerability is higher (19, 20). Concerning HLA-DP, a non-permissive mismatch has been reported to induce adverse effects, depending on the overall number of mismatches and its association with other HLA mismatches (37–39). We hypothesized that a higher class II HED could be associated with an increase in the allo-antigens presented by the host’s antigen-presenting cells. However, no significant association between GVHD and class II HED was found. The negative impact of class II HED, and particularly HLA-DQB1 remains to be clarified.

Overall, the present study introduces HED as an original and relevant marker in HCT. As a surrogate marker of the immunopeptidome presented by leukemic cells, HED assessment could be of particular interest for evaluating alloreactivity in HCT donors. Moreover, the lack of association between class I/class II HED ratio and GVHD could be in favor of a specific GVL marker. Broader studies are needed to confirm the positive impact of class I HED and better evaluate its effect in other malignant diseases.

## Data Availability Statement

The raw data supporting the conclusions of this article are available from the corresponding author upon reasonable request.

## Ethics Statement

The study was performed according to the local ethics committee guidelines (ID: 21_5581). The patients/participants provided their written informed consent to participate in this study.

## Author Contributions

A-MD: Conceptualization, data curation, investigation, methodology, resources, writing—original draft. VD: Resources, validation, writing—review and editing. HL-W: Resources, validation, writing—review and editing. FV, FB, SD-L, M-VL, MB, LG, GF, HG, MH: Resources, validation. FA: Supervision, writing—review and editing. VA: Conceptualization, supervision, data curation, formal analysis, methodology, investigation, resources, validation, visualization, writing—original draft. All authors contributed to the article and approved the submitted version.

## Conflict of Interest

The authors declare that the research was conducted in the absence of any commercial or financial relationships that could be construed as a potential conflict of interest.

## Publisher’s Note

All claims expressed in this article are solely those of the authors and do not necessarily represent those of their affiliated organizations, or those of the publisher, the editors and the reviewers. Any product that may be evaluated in this article, or claim that may be made by its manufacturer, is not guaranteed or endorsed by the publisher.
