# Supplementary material for: Class I/Class II HLA Evolutionary Divergence Ratio Is an Independent Marker Associated With Disease-Free and Overall Survival After Allogeneic Hematopoietic Stem Cell Transplantation for Acute Myeloid Leukemia
Source: Front Immunol. 2022 Mar 4;13:841470. doi: 10.3389/fimmu.2022.841470 (PMC8931406; doi:10.3389/fimmu.2022.841470)
Supplement: Supplementary file 1 [file DataSheet_1.docx]

**SUPPLEMENTARY DATA**

**Supplementary methods**

**Immune parameters**

Lymphocytes subsets were identified by flow cytometry using (with an Navios Beckman Coulter flow cytometer) the following markers : CD45RA+CCR7- for naïve, CD45RA-CCR7+ for central memory (_CM_), CD45RA-CCR7- for effector memory (_EM_) and CD45RA+CCR7- for terminal effector memory (_TE_) T-cells among CD3+ and CD4+ or CD8+ cells; CD19+ and CD20+ for B cells among CD3- lymphocytes, and CD16 and/or CD56+ NK cells among CD3- lymphocytes. Results are expressed as numbers of lymphocytes per µL.

**Supplementary Figure S1. Mean HED scores in AML patients and alleles’ HED.**

A: Overall workflow of HED calculation. Protein sequences corresponding to the peptide-binding domain (exons 2 and 3 for class I and exon 2 for class II) of each HLA molecule referenced in the IMGT HLA database (14) is extracted using Ensembl annotations (15). Divergences between each allele is calculated using the Grantham distance. Mean HED scores are then calculated in both donors and recipients as the mean divergence between the two copies of HLA-A, -B, and -C for class I, and HLA-DRB1, -DPB1, and -DQB1 for class II.

B: Boxplots representing mean recipient (R) and donor (D) HED scores in AML patients.

C: Correlations between HED scores of each allele in AML patients. For each allele, pearson’s correlation coefficient between recipients (rows) and donors (columns) are represented with FDR-adjusted p-values significance (*: <0.05, **:<0.01, ***:<0.001).

D: Correlations between donor and recipients HED scores according to HLA matching. Pearson’s correlation coefficients between donors and recipients’ HED scores are represented with FDR-adjusted p-values significance (*: <0.05, **:<0.01, ***:<0.001).

*Abbreviations: AML, acute myeloid leukemia; HED, HLA evolutionary divergence.*

**Supplementary Figure S2. Multivariate analysis of class I / class II HED ratio association with GVHD, neutrophil, and platelet recovery.**

A: Association between individual class I and II HED scores and class I / class II HED ratio with aGVHD (left) and severe grade 3/4 aGVHD (right). Hazard ratio (HR) were computed using a multivariate cox model controlling for disease status, HLA matching, conditioning, and graft source.

B: Association between individual class I HED scores and cGVHD (left) and extensive cGVHD (right). HR were computed using a multivariate cox model controlling for disease status, HLA matching, conditioning, and graft source.

C: Association between individual class I HED scores and neutrophils (>0,5 G/L; left) and platelets (>20G/L; right). HR were computed using a multivariate cox model controlling for disease status, HLA matching, conditioning, and graft source.

*Abbreviations: aGVHD, acute graft versus host disease; cGVHD, chronic graft versus host disease; CI, confidence interval; HR, hazard ratio; HED, HLA evolutionary divergence; R, recipient.*
