# Supplementary figures and images for: Class I/Class II HLA Evolutionary Divergence Ratio Is an Independent Marker Associated With Disease-Free and Overall Survival After Allogeneic Hematopoietic Stem Cell Transplantation for Acute Myeloid Leukemia
Source: Front Immunol. 2022 Mar 4;13:841470. doi: 10.3389/fimmu.2022.841470 (PMC8931406; doi:10.3389/fimmu.2022.841470)

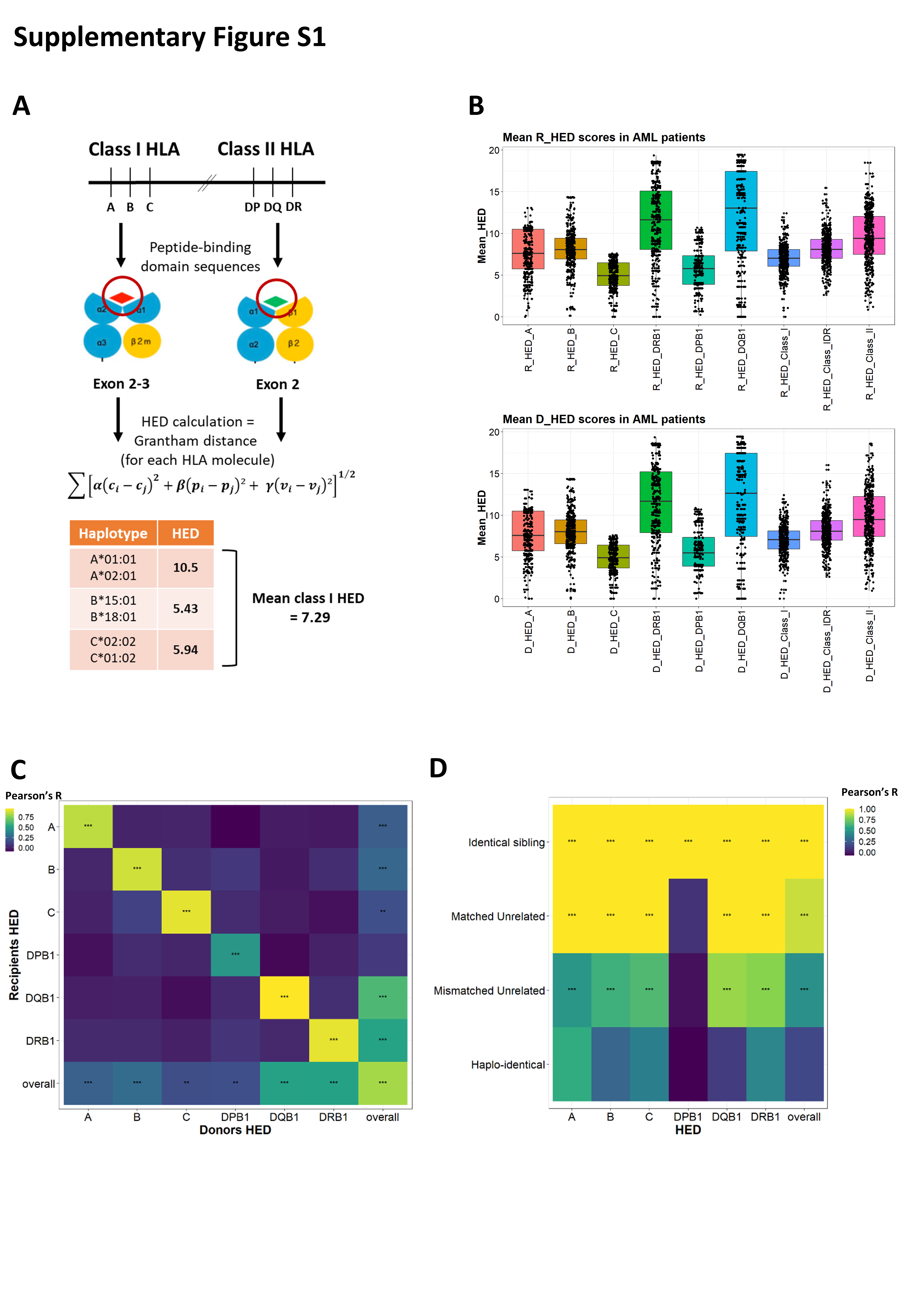

Supplement: Supplementary file 2 [file Image_1.tif]

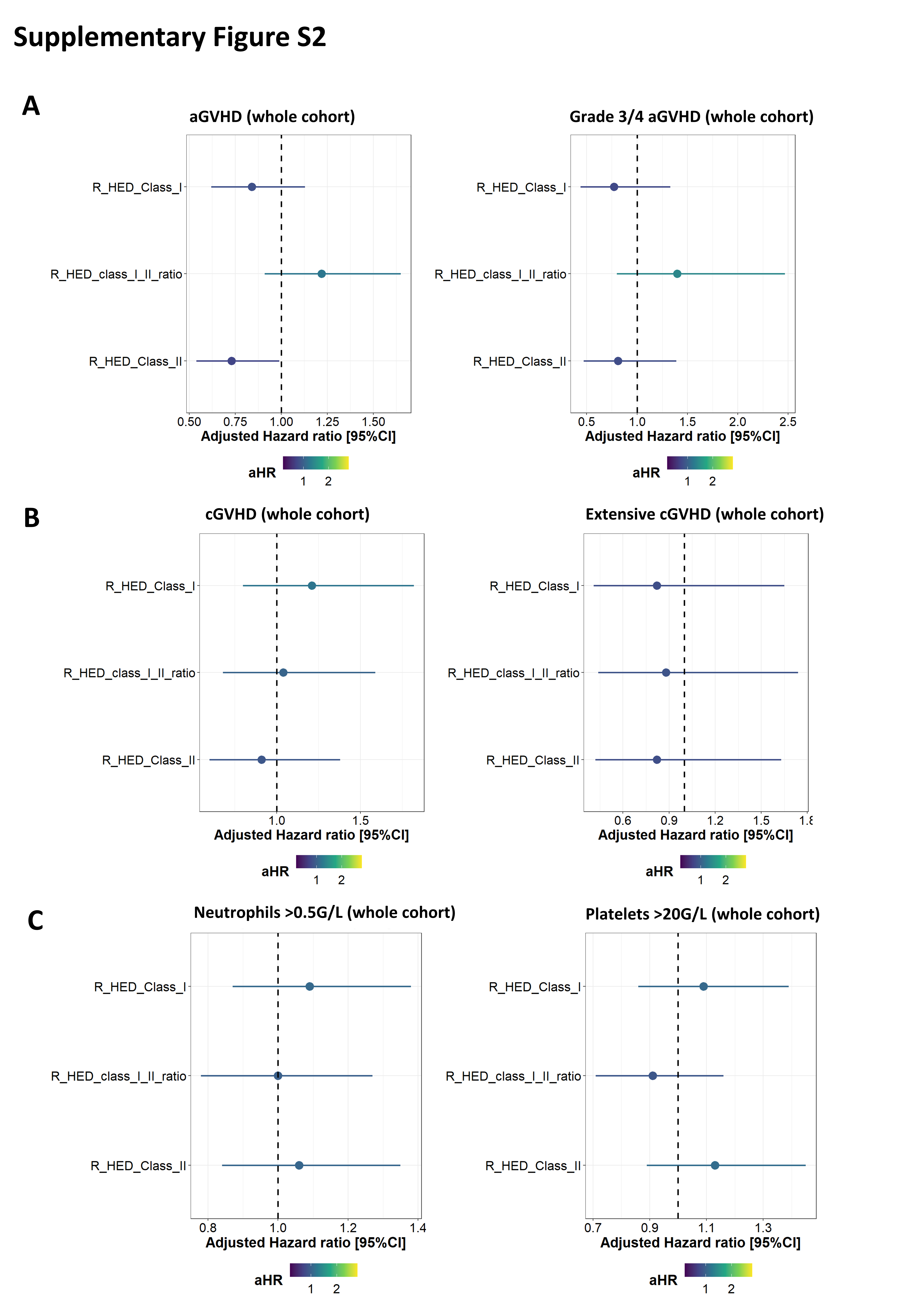

Supplement: Supplementary file 3 [file Image_2.tif]
